# The Effects of Caffeine on Jumping Performance and Maximal Strength in Female Collegiate Athletes

**DOI:** 10.3390/nu13082496

**Published:** 2021-07-22

**Authors:** Benjamin I. Burke, S. Kyle Travis, Jeremy A. Gentles, Kimitake Sato, Henry M. Lang, Caleb D. Bazyler

**Affiliations:** 1 Center of Excellence for Sport Science and Coach Education, Department of Sport, Exercise, Recreation and Kinesiology, East Tennessee State University, Johnson City, TN 37614, USA; travissk@etsu.edu (S.K.T.); gentlesj@etsu.edu (J.A.G.); bazylerc@etsu.edu (C.D.B.); 2 Peak Force, International, Inc., Taichung 42151, Taiwan; ceo@peakforce.cc; 3 Department of Exercise Physiology, University of Mary, Bismarck, ND 58504, USA; langhm@etsu.edu

**Keywords:** methylxanthine, ergogenic substances, neuromuscular performance, force production, resistance training, anaerobic exercise

## Abstract

Caffeine is often used in a variety of forms to enhance athletic performance; however, research regarding caffeine’s effects on strength and power in female athletes is lacking. Therefore, the purpose of this study was to analyze the acute effects of caffeine anhydrous (6 mg/kg of body mass) on jumping performance and maximal strength in female collegiate athletes. Eleven athletes (19.7 ± 0.9 yrs; 166.4 ± 10.2 cm, 67.7 ± 9.4 kg) performed two testing sessions separated by one week, and randomly received caffeine or placebo using a double-blind approach. Heart rate, blood pressure, and tympanic temperature were recorded before athletes received each condition, following 60 min of quiet sitting, and directly after performance testing. Athletes were assessed on unweighted and weighted squat jump height (SJH0, SJH20) and countermovement jump height (CMJH0, CMJH20), isometric mid-thigh pull peak force (IPF), and rate of force development from 0–200 ms (RFD200). Resting systolic blood pressure was significantly greater following caffeine administration compared to a placebo (*p* = 0.017). There were small, significant differences in SJH0 (*p* = 0.035, g = 0.35), SJH20 (*p* = 0.002, g = 0.49), CMJH0 (*p* = 0.015, g = 0.19), and CMJH20 (*p* < 0.001, g = 0.37) in favor of caffeine over placebo. However, there was no significant difference in IPF (*p* = 0.369, g = 0.12) and RFD200 (*p* = 0.235, g = 0.32) between conditions. Therefore, caffeine appears to enhance jumping performance, but not maximal strength in female collegiate athletes.

## 1. Introduction

Caffeine, the world’s most consumed psychoactive drug, has a number of positive physiological effects linked to increasing aerobic and anaerobic performance [1,2,3,4]. The mechanisms behind these effects in anaerobic performances have not been definitively established, although there are several hypotheses. Caffeine has been shown to increase catecholamines such as epinephrine, leading to increased central nervous system (CNS) stimulation, possibly contributing to enhanced performance [1,2]. Caffeine has also been shown to increase the release of calcium from the sarcoplasmic reticulum, thus leading to enhanced force production [3]. Caffeine supplementation may also increase body temperature [5], which influences muscle contractility [6]. Therefore, caffeine can enhance motor unit recruitment and rate coding of large muscle groups, possibly explaining increases in performance [7,8]. One prominent hypothesis for caffeine’s ergogenic effects concerns its action on adenosine. Adenosine is a neurotransmitter that acts as a CNS depressant, eliciting multiple cognitive effects such as decreased arousal that could interfere with physical exertion [4]. Caffeine acts as a non-selective adenosine inhibitor, blunting the negative effects of adenosine on performance and stimulating the CNS [4].

A plethora of studies have examined the effects of caffeine on endurance exercise, leading to the general conclusion that caffeine enhances performance in aerobic activities [9,10,11,12,13]. Ivy et al. [9] reported a 7.4% increase in work production in trained cyclists over the course of a two hour cycling session after receiving two doses of caffeine at 250 mg each compared to a control group. Additionally, Denadai and Denadai [10] found that time to exhaustion for untrained males on a cycle ergometer was significantly greater with a 5 mg/kg dose of caffeine compared to a placebo. Reviews and meta-analyses spanning the last two decades have also concluded that caffeine improves endurance performance [11,12,13]. Thus, due to these ergogenic effects, caffeine is commonly used by endurance athletes.

Conversely, fewer studies have examined the effects of caffeine on anaerobic performance, which has produced conflicting results. Arazi et al. [14] reported a significant increase in maximal bench press and leg press strength following the consumption of caffeine by novice male bodybuilders (6 mg/kg). However, Astorino et al. [15] found no effect when examining the same lifts and dosage in resistance trained men. Beck et al. [16] also recorded increases in upper body maximal strength in resistance trained men yet found no change in lower body maximal strength. These findings were confirmed by a meta-analysis examining 10 studies that concluded that caffeine-induced increases in maximal strength appeared to be limited to the upper body [17]. 

Furthermore, multiple studies reported increased power performance (e.g., jump height, peak power) relative to caffeine consumption [7,18]. For instance, a meta-analysis by Grigic et al. [17] concluded that caffeine acutely increased vertical jump height in a mixed subject population (i.e., trained, untrained) compared to a placebo. However, Williams et al. [19] noted a 350 mg dose of caffeine did not increase peak or mean power in resistance trained men performing the Wingate power test. Thus, further research is needed assessing the effects of caffeine on power performance.

Males and females may exhibit different responses to caffeine, such as physiological alterations and performance outcomes due to differences in circulating steroid hormones [20]. Studies examining the effects of caffeine on maximal strength and power in female subjects are also limited and conflicting [18]. Indeed, multiple researchers have highlighted the need for studies examining the effects of caffeine on performance in females [17,21,22]. Ali et al. [18] showed that a 6 mg/kg dose of caffeine improved eccentric strength during isokinetic dynamometry, yet Sabblah et al. [23] showed no increase in 1-repetition maximum (1RM) squat performance in females following a 5 mg/kg dose of caffeine compared to a placebo. Since single-joint evaluations of strength are less applicable to anaerobically dominant female athletes compared with multi-joint, lower body strength tests [24], most studies assess maximal strength using 1RM testing [17,21,23]. However, multi-joint isometric testing with force platforms (e.g., isometric mid-thigh pull, isometric squat) may provide additional sensitivity and enhance the ability to detect differences between caffeine and placebo [25]. Notably, only one study has examined the effects of caffeine on strength performance using the isometric mid-thigh pull, albeit with recreationally trained females [26]. As for maximal power, several studies examined the effects of caffeine on power output in females using various methodology (e.g., vertical jumps, Wingate) [18,22,26,27], with very few finding an increase in power output [18].

Importantly, to the best of our knowledge, only three studies have examined the effects of caffeine on anaerobic performance in female athletes [18,22,27]. These studies were limited by the use of tests with poor external validity (e.g., isokinetic dynamometry), inclusion of females using oral-contraceptive steroids, which have been shown to interfere with caffeine metabolism [18], and lack of physiological measures (e.g., heart rate, blood pressure, temperature). Goldstein et al. [21] demonstrated a different physiological response to caffeine in females compared to previous investigations with males [15]. These findings were later confirmed by Temple et al. [20], furthering the need for additional examinations of females’ physiological response to caffeine. Furthermore, a review by Grgic and Del Coso [28] concluded that the assessment of lower body strength with females implementing caffeine supplementation is warranted due to the equivocal findings in the literature thus far. Due to these limitations, further research is needed assessing the effects of caffeine on multi-joint, lower body strength and power in female athletes. Therefore, the primary aims of this study were to analyze the acute effects of 6 mg/kg caffeine, a dose that has been shown to maximize physiological responses to caffeine [29], on (1) jumping performance and (2) lower body maximal strength in female collegiate athletes. Secondarily, this study aimed to assess the physiological response to caffeine ingestion compared to a placebo. We hypothesized that ingestion of caffeine would significantly increase strength and jumping performance, and elicit a greater physiological response compared to a placebo.

## 2. Materials and Methods

### 2.1. Experimental Approach

A randomized, double-blind, placebo-controlled, crossover design was used for this study. Athletes received either a placebo or a 6 mg/kg dose of caffeine anhydrous 60 min prior to testing. This dosage has been shown to maximize caffeine’s effectiveness [29] and optimize blood-caffeine levels within 60 min [30]. Caffeine was consumed in pill form along with 250 mL of water. The placebo, sucralose, was administered in the same fashion. Testing was conducted in the same environmental conditions at the same time of day and day of the week for both sessions.

Athletes were required to avoid strenuous activity and the ingestion of caffeine or caffeine-containing foods, a list of which were discussed, for 36 h preceding each of the two testing sessions. The subjects kept a dietary log for 48 h before the first testing session and were asked to replicate their diet prior to the second session. Athletes also self-reported oral contraceptive steroid use and menstrual cycle phase due to their potential effects on caffeine metabolism [17,18,31,32]. Following an overnight fast, athletes arrived at the laboratory in a rested and hydrated state. Prior to the first testing session, athletes completed a questionnaire addressing their health history, menstrual cycle phase, habitual caffeine ingestion, sleep duration, and sport-related information (i.e., position, years on the team, number of previous testing sessions performed). A second questionnaire was then completed assessing subjective stress and recovery states using the short recovery and stress scale (SRSS) [33]. Physiological measurements were assessed after the second questionnaire, followed by the administration of the caffeine or placebo. After 60 min of quiet sitting, physiological measurements were reassessed. Athletes proceeded to complete a dynamic warm-up followed by vertical jumps and isometric mid-thigh pulls. One week later, the athletes repeated the same testing procedures with the opposite condition. To assess the effectiveness of blinding, the athletes were asked if they could identify whether they received the caffeine supplement or placebo.

### 2.2. Participants

Eleven female collegiate volleyball players (*n* = 8) and weightlifters (*n* = 3) participated in this study (Table 1). The athletes regularly performed the tests conducted in this study as part of an on-going athlete monitoring program. However, to ensure adequate familiarization, only athletes who had completed at least two prior testing sessions as part of the monitoring program were eligible to participate. Any athlete under the age of 18 or with lower body injuries less than 3 months prior to testing was excluded from the study. Prior to data collection, athletes received information about the purpose of the study and provided written informed consent. The study received approval from the university’s institutional review board (IRB# 0919.23s).

### 2.3. Procedures

#### 2.3.1. Randomization

An assistant, unaffiliated with the study, used a coin flip to randomly assign the condition. Each participant received a randomized condition during session one and received the opposite condition during session two. Every procedure detailed below was repeated during sessions one and two.

#### 2.3.2. Anthropometrics and Hydration

Upon arrival to the laboratory, hydration was evaluated prior to testing using a refractometer (ATOGO, Tokyo, Japan). The athletes were considered hydrated if urine specific gravity (USG) was <1.020. If an athlete failed testing, they continued to consume water until passing the test. A calibrated scale (Tanita BF-350, Arlington Heights, IL, USA) was used to measure body mass to the nearest 0.1 kg. Height measurements were recorded to the nearest 0.5 cm using a stadiometer (Cardinal Scale Manufacturing Co., Webb City, MO, USA).

#### 2.3.3. Questionnaires

The questionnaires were administered during the five minutes of quiet sitting following anthropometrics and hydration. The first questionnaire asked participants to report their health history, menstrual cycle phase, habitual caffeine ingestion, sleep duration, and sport-related information, while the second assessed overall stress and overall recovery state using the SRSS [33].

#### 2.3.4. Condition Administration and Physiological Measurements

Upon completing hydration testing, athletes sat quietly for five minutes. After five minutes of quiet sitting a technician took each athlete’s heart rate (HR), blood pressure (BP) (Omron Blood Pressure Monitor, Lake Forest, IL, USA), and tympanic temperature (TT) (Braun ThermoScan 5, Kronberg, Germany) (T1). The placebo (Splenda, Heartland Food Products Group, Carmel, IN, USA) or caffeine (W222402-1KG-K, Sigma-Aldrich, Saint Louis, MO, USA) supplement was then administered in pill form, followed by 60 min of quiet sitting. At the end of this period, HR, BP, and TT were recorded again (T2). Within 60 s of completing performance testing, HR, BP, TT, and session rating of perceived exertion (RPE, 1–10 scale) were recorded in the same seated position (T3).

#### 2.3.5. Standardized Warm-Up

Each athlete performed a standardized warm-up consisting of 25 jumping-jacks, 5 repetitions of mid-thigh pulls with a 20 kg barbell, and 3 sets of 5 repetitions of mid-thigh pulls with 40 kg. Each set was followed by 60 s of rest.

#### 2.3.6. Vertical Jumps

**Figure 1:**
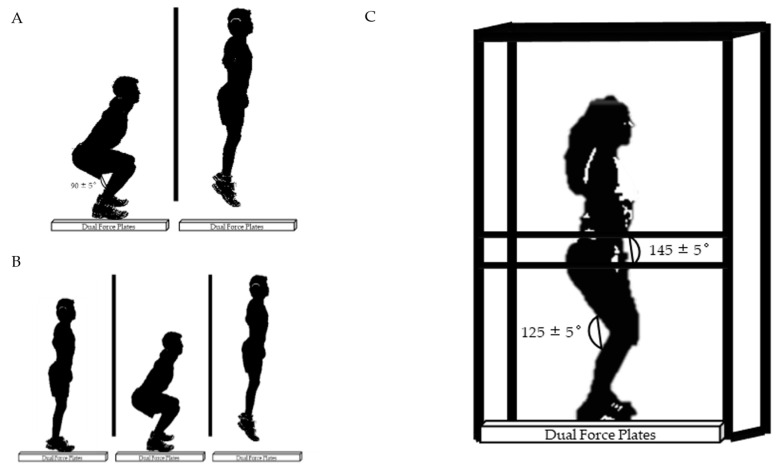
Performance testing. Panel (**A**) depicts the start and flight phase of squat jump testing, panel (**B**) depicts countermovement jump testing, and panel (**C**) depicts the isometric mid-thigh pull positioning.

Upon completing the standardized warm-up, athletes were given two minutes of rest and then proceeded to vertical jump testing. The athletes performed squat jumps (SJ) followed by countermovement jumps (CMJ) (Figure 1). All jumps were completed on dual force plates (Rice Lake Weighing Systems, Rice Lake, WI, USA; 1000 Hz sampling rate) placed on a 91.4 cm × 91.4 cm custom built jumping platform [34]. For unweighted jumps, athletes placed a near weightless (<1 kg) polyvinyl chloride (PVC) pipe on their shoulders to eliminate the use of arm swing while jumping. For the weighted jumps, a 20 kg bar was used. Athletes were told to squat down to the “ready position,” a 90° knee angle measured with a handheld goniometer. After hearing the command of “3, 2, 1, jump!”, they gave a maximum effort jump. Warm-ups for SJ were performed at 50% and 75% perceived effort prior to at least two maximal effort jumps. The athletes then moved on to the CMJ test where they were instructed to stand still and await the command of “3, 2, 1, jump!” before descending to a self-selected depth and jumping. CMJ at 75% perceived effort preceded the two maximal effort trials with a 60 s rest in between. If the difference in jump height was greater than 2 cm, additional trials were performed.

Loaded and unloaded squat jump height (SJH0, SJH20) and countermovement jump height (CMJH0, CMJH20) were recorded and analyzed using a custom analysis program (LabView 2010, National Instruments Co., Austin, TX, USA). The mean of two trials’ <2 cm difference in jump height was used for analysis. Jump height was estimated from flight time as described by Linthorne [35]. Test–retest reliability has been reported previously for jump height from our laboratory (intraclass correlation coefficient (ICC) = 0.98, and coefficient of variation (CV) = 2.20%) [36,37].

#### 2.3.7. Isometric Mid-Thigh Pulls

After completing SJ testing, athletes performed isometric mid-thigh pulls on dual force plates (Rice Lake Weighing Systems, Rice Lake, WI, USA; 1000 Hz sampling rate) covering an area of 91.4 cm × 91.4 cm (Figure 1) [34]. The isometric mid-thigh pulls were performed inside of a custom designed power rack with a fixed bar that was adjusted to suit each athlete’s mid-thigh position. The athletes were put into their respective pulling positions while remaining upright with a knee angle of 125 ± 5° and hip angle of 145 ± 5° measured with a handheld goniometer. The athletes were then secured to the bar in their respective power clean grip positions using lifting straps and athletic tape to control for grip strength (Figure 1). The athletes were given the command “steady tension on the bar”, indicating the need to remain stable. The tester then shouted “3, 2, 1, pull!” and athletes pulled until peak force was reached and the tester told them to stop. Warm-up attempts at 50% and 75% perceived effort were performed preceding maximal effort attempts. For the maximal effort pulls, the athletes received loud, verbal encouragement and were instructed to pull as “fast and hard” as possible. If a countermovement on the force–time curve of >200 N was detected, the athlete was given another attempt. The test was terminated if a consistent decrease or plateau in peak force was observed. An additional trial was also administered if a difference in peak force of >250 N between the first two trials. Rest periods of three minutes were allotted between attempts.

All isometric peak force (IPF) values reported were gross values not offset by the athlete’s body mass on the force plate and were subsequently allometrically scaled for body mass (IPFa) [38]. Average rate of force development (RFD) from 0 to 200 ms (RFD200) was considered for the analysis. Analog data from the force plate were amplified and conditioned (low pass at 16 Hz) with a Transducer Techniques amplifier and conditioning module (Temecula, CA, USA). An analog–digital converter (DAQCard-6063E, National Instruments, Austin, TX, USA) was used for collection at 1000 Hz. A custom analysis program (LabView 2010, National Instruments Co., Austin, TX, USA) was used to analyze the mean of two trials within 250 N. Test–retest reliability has been previously reported for IPF and RFD from our laboratory (ICC = 0.95, 0.83, CV = 2.83%, 12.01%, respectively) [39].

### 2.4. Statistics

The dataset was initially screened for outliers (mean ± 3 SD) followed by a Shapiro–Wilks test to assess normality and Mauchly’s test to assess sphericity. A paired samples t-test was used to compare performance results between conditions. A repeated measures ANOVA followed by post-hoc comparisons was used to analyze changes in physiological measures between and within conditions. Alpha level for all analyses were set at *p* < 0.05 and a Benjamini–Hochberg adjustment was used to correct for multiple comparisons [40]. Effect sizes were determined using Hedge’s g_av_ with 95% confidence intervals (CIs), and classified using the following scale: <0.2 (trivial); 0.2–0.6 (small); 0.6–1.2 (moderate); 1.2–2.0 (large); 2.0–4.0 (very large); >4.0 (nearly perfect) [41]. Individual changes in performance measures following caffeine administration were considered meaningful if they exceeded the typical error. A power analysis conducted with G*POWER 3.1 (Universität Kiel, Germany) determined that 34 participants were needed in the present study for a power of 0.80, with an effect size of 0.5 and an α= 0.05. Analyses were performed using SPSS software version 25 (IMB Co., New York, NY, USA), and Microsoft Excel 2016 (Microsoft Corporation, Redmond, WA, USA).

## 3. Results

### 3.1. Hydration and Questionnaires

There were no significant differences between testing sessions for body mass, USG, or any SRSS items. Eight of the eleven participants were able to correctly identify the caffeine condition.

### 3.2. Physiological Measurements

There were statistically significant time effects for HR (F (5, 50) = 10.645, *p* < 0.001,), SBP (F (2.625, 26.248) = 8.414, *p* = 0.001), DBP (F (5, 50) = 3.321, *p* = 0.011), and TT (F (5, 50) = 5.462, *p* < 0.001). Post-hoc analyses revealed statistically significant increases in HR (*p* < 0.001, *p* = 0.003), SBP (*p* = 0.001, *p* = 0.002), DBP (*p* = 0.024, *p* < 0.001), and TT (*p* = 0.039, *p* = 0.003) from T1–T3 in the placebo and caffeine conditions, respectively. There were also statistically significant increases in SBP (*p* < 0.001) and DBP (*p* = 0.009) from T1–T2 in the caffeine condition. SBP was significantly greater in the caffeine condition compared to the placebo at T2 (*p* = 0.017). There were no significant differences in RPE between caffeine and placebo (5.73 ± 1.78 and 5.59 ± 1.46, respectively) (Table 2).

### 3.3. Performance Measurements

**Figure 2:**
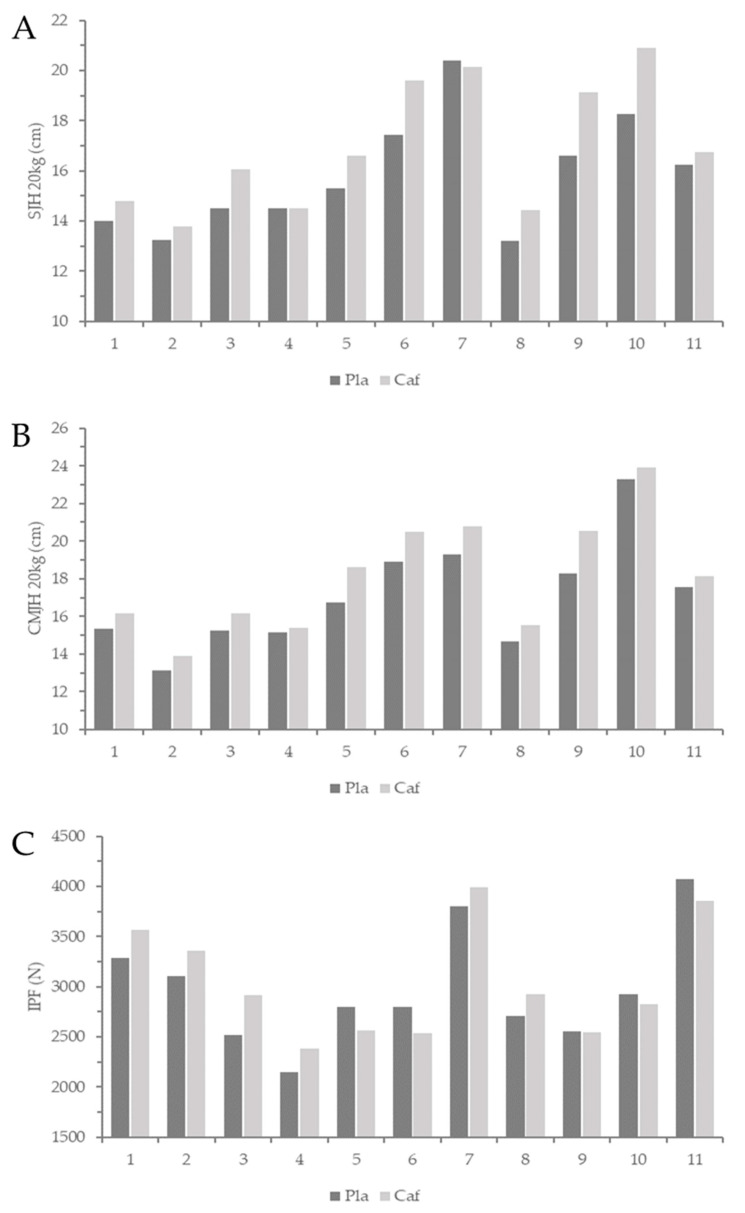
Individual changes in (**A**) SJH 20 kg = squat jump height with 20 kg, (**B**) CMJH 20 kg = countermovement jump height with 20 kg, and (**C**) IPF = isometric mid-thigh pull peak force. Nine of eleven athletes improved SJH 20 kg, 10 of 11 athletes improved CMJH 20 kg, and 6 of 11 athletes improved IPF in the caffeine condition over placebo relative to the typical error for each measurement. Pla = placebo, caf = caffeine.

There were small, statistically significant differences in SJH0 (*p* = 0.035, g = 0.35), SJH20 (*p* = 0.002, g = 0.49), CMJH0 (*p* = 0.015, g = 0.19), and CMJH20 (*p* < 0.001, g = 0.37) in favor of caffeine over the placebo. There were no significant differences in IPF (*p* = 0.369, g = 0.12), IPFa (*p* = 0.20, g = 0.13), and RFD200 (*p* = 0.235, g = 0.32) between caffeine and the placebo (Table 3). Analysis of individual performance changes revealed that 9 of 11 athletes improved SJH 20 kg (+0.5 to 2.7 cm), 10 of 11 athletes improved CMJH 20 kg (+0.8 to 2.3 cm), and 6 of 11 athletes improved IPF (+190.26 to 391.84 N) in the caffeine condition over the placebo relative to the typical error for each measurement (Figure 2).

## 4. Discussion

The main finding of this study was that ingestion of a 6 mg/kg dose of caffeine has a small, significant effect on jumping performance in female collegiate athletes. However, maximal isometric strength was not significantly affected by caffeine consumption. The only physiological measurement that was significantly altered by caffeine compared to the placebo was resting SBP. There were no significant changes in RPE between conditions. These results support our hypothesis that caffeine would enhance jumping performance; however, the results failed to show a significant increase in maximal strength, and the difference in physiological response was minimal compared to the placebo.

The increases in jumping performance recorded in the present study are consistent with previous studies [7,42]. Bloms et al. [7] found statistically significant increases in SJH and CMJH, peak force, and average RFD in male and female collegiate athletes following 5 mg/kg of caffeine compared to a placebo. Diaz-Lara et al. [42] also recorded increased CMJ height and velocity at peak power in male Jiu-jitsu athletes following the ingestion of 3 mg/kg of caffeine. Furthermore, a meta-analysis by Grgic et al. [17] concluded that acute caffeine ingestion can be effective for enhancing jumping performance.

However, the current study is the first to show caffeine’s benefits on jumping performance in a strictly female athlete sample. Notably, 10 of 11 athletes improved loaded CMJH in the caffeine condition (+0.8 to 2.3 cm), indicating a consistent beneficial effect over placebo. Ali et al. [18] showed no significant differences in CMJH or estimated leg power in female athletes following 6 mg/kg of caffeine ingestion when compared to a placebo. In agreement, Arazi et al. [27] failed to show improvements in the Sargent vertical jump test in teenage female karate athletes following 2 or 5 mg/kg of caffeine compared to a placebo. However, Ali et al. [18] only examined female athletes using oral contraceptive steroids, which have been shown to significantly extend the elimination half-life of caffeine and may have influenced the results [18,31,32,43]. Furthermore, Arazi et al. [27] performed jump testing after 1RM testing and muscular endurance testing, in which repetitions with 60% of 1RM were performed until volitional failure. Although testing order was controlled between conditions, it is possible athletes’ jumping performance was compromised due to accumulative fatigue.

This investigation is also the first to examine caffeine’s effects on isometric mid-thigh pull performance in female collegiate athletes. Based on our findings, no increases were observed in the caffeine condition compared to a placebo. In contrast to jumping performance, only 6 of 11 athletes improved IPF (+190.26 to 391.84 N) in the caffeine condition over placebo. However, other studies have investigated strength performance using different methodologies [18,27]. Ali et al. [18] used isokinetic dynamometry, finding increases in eccentric knee flexion after the ingestion of 6 mg/kg of caffeine. Arazi et al. [27], on the other hand, found no increase in strength performance when using a 1RM leg press test to evaluate caffeine’s effects on strength performance. Nonetheless, Arazi et al. [27] only included teenage female athletes, who had comparatively less training experience. Considering caffeine may have larger ergogenic effects on trained muscle [15,22,44], this could partly explain the discrepancy between Ali et al. [18] and Arazi et al. [27] in caffeine’s effects on strength performance. Meta-analytic results suggest caffeine’s effect on maximal strength performance is small (effect size ~0.20) compared to a placebo [17,28]. Thus, with a sample size of 11 athletes, it is possible our study was underpowered. Nonetheless, our results agree with the meta-analyses by Grgic et al. [17] and Grgic and Del Coso [28] showing no effect of caffeine on lower-body maximal strength.

Caffeine has been shown to illicit different physiological responses in females compared to males [20,21]. A statistically significant difference in physiological measurements between conditions occurred only in resting SBP at T2 in favor of caffeine. These findings agree with Goldstein et al. [21] who found SBP to be significantly affected by caffeine, while HR and DBP remained unchanged. However, Goldstein et al. [21] found SBP was only significantly altered in the caffeine condition after testing, while the current study found differences only at rest after caffeine administration. In contrast, Astorino et al. [15] found caffeine affected multiple physiological variables (HR, SBP) when compared with a placebo in males. Temple et al. [20] found that females exhibited greater changes in blood pressure but not in heart rate compared to males, possibly explaining the difference between the aforementioned studies [15,21]. Differences in physiological responses may also be due to inter-individual differences in caffeine habituation or metabolism [20,21,45]. Future studies should examine the influence of caffeine habituation and individual caffeine metabolism on strength and power performance, specifically in female athletes. No statistically significant differences in RPE were detected between conditions. Although some studies have shown caffeine to reduce RPE when compared with a placebo [27], our findings agree with previous literature suggesting that RPE is inadequate in detecting changes in perception at very high intensities [4].

In regard to physiological mechanisms, the increases in jumping performance following caffeine ingestion observed in this investigation could be due to enhanced motor unit recruitment and rate coding of large, lower-body muscle groups through caffeine’s excitatory effect at the supraspinal level [7,8]. Furthermore, caffeine blunts pain perception during exercise through its action on adenosine receptors, which may increase maximal voluntary contractile force [4]. Therefore, caffeine’s action as an adenosine antagonist remains a strong prospect to explain caffeine’s effects on jumping performance in this investigation. Caffeine has also been shown to increase alertness, decrease reaction time, and possibly improve logical reasoning, all of which may lead to increase performance in sport as well as laboratory testing [46]. Nonetheless, 8 of 11 athletes correctly identified the caffeine condition, suggesting inadequate blinding may have influenced our results. While difficult in practice, future investigations should develop novel strategies to control for blinding in caffeine research.

The findings presented in this study are relevant to female athletes whose sports involve high force output and power production, especially for sports that include jumping (e.g., volleyball, basketball). However, there are several limitations to the current study. Among these limitations were a small sample size, an inability to adequately blind the conditions, and the examination of a single dosage of caffeine. Furthermore, the current study did not control for inter-individual differences in caffeine habituation or metabolism. Future research should address these issues by amassing a larger sample size, determining ways to adequately blind caffeine, examining multiple doses of caffeine (e.g., 3 or 5 mg/kg), and controlling for high vs. low responders to caffeine.

## 5. Conclusions

Despite the prevalent use of caffeine as an ergogenic aid in athletics, very little research has examined the effects of caffeine on strength and power in female athletes. Our findings indicate that a 6 mg/kg dose of caffeine has a small, significant effect on jumping performance in female collegiate athletes. These effects were not observed in measures of maximal strength. The only physiological measurement that was significantly altered by caffeine compared to the placebo was resting SBP. Future research should aim to examine the acute effects of caffeine ingestion on maximal strength and jumping performance in a larger sample of female athletes, and develop novel strategies to control for blinding.

## Figures and Tables

**Table 1:** Participant demographics.

Title 1	Mean	Range
Age (yrs)	19.7 ± 0.9	18.1–21.3
Height (cm)	166.4 ± 10.2	149.0–178.0
Mass (kg)	67.7 ± 9.4	54.1–85.0
Experience (yrs) *	1.9 ± 1.2	1.0–5.0
Daily Caffeine Intake (mg) ^#^	90.4 ± 85.7	14.0–312.0

* Years of experience at the collegiate level, ^#^ self-reported.

**Table 2:** The effects of caffeine on physiological measurements.

Physiological Variables			
Placebo Condition	Time Point 1	Time Point 2	Time Point 3
Heart Rate (beats/min)	79 ± 12	75 ± 11	92 ± 12 *
Systolic Blood Pressure (mmHg)	110 ± 8	110 ± 8	121 ± 4 *
Diastolic Blood Pressure (mmHg)	73 ± 5	75 ± 7	77 ± 7 *
Tympanic Temperature (F)	97.7 ± 0.8	97.8 ± 0.6	98.4 ± 0.8 *
**Caffeine Condition**			
Heart Rate (beats/min)	74 ± 11	71 ± 6	91 ± 14 *
Systolic Blood Pressure (mmHg)	108 ± 6	117 ± 9 *^,#^	122 ± 10 *
Diastolic Blood Pressure (mmHg)	71 ± 6	76 ± 7 *	79 ± 8 *
Tympanic Temperature (F)	97.5 ± 0.9	97.6 ± 0.8	98.5 ± 1.0 *

* Significant increase compared to Time Point 1 (*p* < 0.05), ^#^ Significant increase compared to the placebo (*p* < 0.05).

**Table 3:** The effects of caffeine on performance parameters.

Performance Variables	Placebo	Caffeine	*p*-Value	Hedge’s *g* [95% CI]	Typical Error
SJH 0 kg (cm)	23.49 ± 3.15	24.55 ± 2.86	0.035 *	0.35 [0.08, 0.63]	1.02
SJH 20 kg (cm)	15.79 ± 2.26	16.98 ± 2.56	0.002 *	0.49 [0.25, 0.74]	0.69
CMJH 0 kg (cm)	25.30 ± 3.77	26.00 ± 3.23	0.015 *	0.19 [0.07, 0.33]	0.55
CMJH 20 kg (cm)	17.05 ± 2.84	18.15 ± 3.03	<0.001 *	0.37 [0.24, 0.50]	0.43
IPF (N)	2976.15 ± 566.13	3043.76 ± 565.06	0.369	0.12 [−0.07, 0.31]	168.57
IPFa (N/kg ^0.67^)	177.68 ± 31.46	181.49 ± 28.17	0.2	0.13 [−0.08, 0.34]	10.32
RFD 200 ms (N·s^−1^)	4921.85 ± 1693.07	5455.67 ± 1596.08	0.235	0.32 [−0.10, 0.75]	990.63

* Significant difference between conditions (*p* < 0.05); SJH = squat jump height; CMJH = countermovement jump height; IPF = isometric peak force; IPFa = allometrically scaled isometric peak force; RFD = rate of force development; CI = confidence intervals.

## Data Availability

The data presented in this study are available on request from the corresponding author.
